# Bis(2,4-dioxo­pentan-3-ido-κ^2^ *O*,*O*′)dioxidomolyb­denum(VI): a redetermination

**DOI:** 10.1107/S2414314621007781

**Published:** 2021-08-06

**Authors:** Dean H. Johnston, Calvin King, Aileen Seitz, Mia Sethi

**Affiliations:** a Department of Chemistry, Otterbein University, Westerville, OH 43081, USA; Vienna University of Technology, Austria

**Keywords:** crystal structure, molybdenum, oxo, acetyl­acetonate, acac

## Abstract

The title compound, [Mo(C_5_H_7_O_2_)_2_O_2_] or *cis*-[MoO_2_(acac)_2_] (acac is acetyl­acetonate), contains a molybdenum(VI) atom coordinated by two acetyl­acetonate ligands and two doubly bonded oxido ligands in a distorted octa­hedral shape. The mol­ecule is chiral and the asymmetric unit contains two independent mol­ecules (one Δ, one Λ). Extensive C—H⋯O contacts are present throughout the structure. Data were collected at 100 K, providing higher precision of unit-cell parameters and atomic positions than previous determinations [Kamenar *et al.* (1973 ▸). *Cryst. Struct. Commun.* **2**, 41–44.; Krasochka *et al.* (1975). *Zh. Strukt. Khim.* **16**, 696–698].

## Abstract 2

The crystal structure of *cis*-[MoO_2_(acac)_2_] has been redetermined at 100 K, providing a more precise description of the structure including hydrogen atoms and inter­molecular contacts.

## Graphical abstract

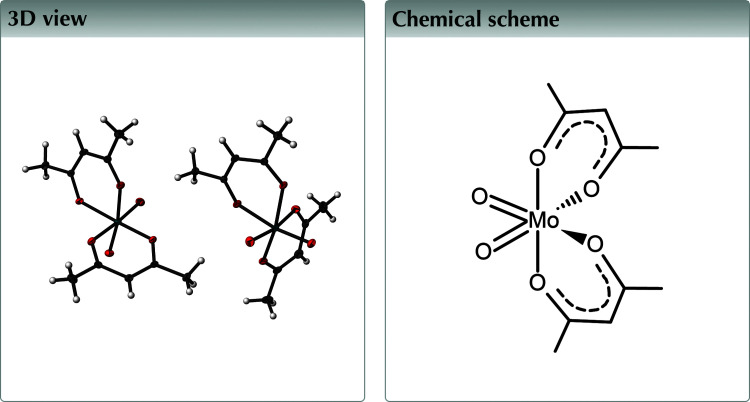

## Structure description

The title compound is a versatile starting material for the preparation of *cis*-dioxidomolybdenum complexes, including complexes containing organodi­nitro­gen ligands (Bustos *et al.*, 1994 ▸) and molybdenyl adducts of platinum *μ*-S dimers (Henderson *et al.*, 2011 ▸). MoO_2_(acac)_2_ has also been used to prepare dioxidomolybdenum(VI) complexes with *O*,*N*,*N*′ chelating ligands (Ceylan *et al.*, 2015 ▸) and an amine bis­(phenolate) ligand (Bowen & Wile, 2021 ▸). Many of these complexes have been prepared and studied for their catalytic activities, including complexes with acyl­pyrazolo­nate ligands that catalyze the de­oxy­genation of epoxides (Hills *et al.*, 2013 ▸; Begines *et al.*, 2018 ▸) and dioxidomol­yb­denum(VI) complexes with salicyl­amide ligands for the epoxidation of olefins (Annese *et al.*, 2019 ▸). Molybdenum(VI) dioxido complexes with acetyl­acetonato ligands have also been investigated for their catalytic properties in the de­hydrogenation of alcohols (Korstanje *et al.*, 2013 ▸). These complexes are of particular inter­est due to their close structural similarities to the active sites of several molybdoenyzmes such as sulfite oxidase, xanthine oxidase, and DMSO reductase (Sousa & Fernandes, 2015 ▸).

Two previous structural determinations of *cis*-dioxidobis(acetyl­acetonato)molybdenum(VI) were published in the mid-1970s (Kamenar *et al.*, 1973 ▸; Krasochka *et al.*, 1975) based on photographic methods and room-temperature data collections. Additionally, Craven *et al.* (1971 ▸) cite an unpublished diffraction study that also confirms the *cis* coordination and includes additional structural information consistent with the current study. None of the previously published structure solutions attempted to locate the positions of any of the hydrogen atoms. Several closely related structures have been determined, including *cis*-dioxido-molybdenum complexes with 1,3-di­phenyl­propane­dianoto ligands (Kojić-Prodić *et al.*, 1974 ▸; Korstanje *et al.*, 2013 ▸) and *tert*-butyl­acetyl­acetonato ligands (Nass *et al.*, 2001 ▸). The structure of the product from the reaction of *cis*-[MoO_2_(acac)_2_] with the strong Lewis acid B(C_6_F_5_)_3_ (Galsworthy *et al.*, 1997 ▸) displays a nearly linear Mo=O—B arrangement [171.2 (1)°] and lengthening of the donating Mo=O bond by about 0.1 Å.

**Figure 1:**
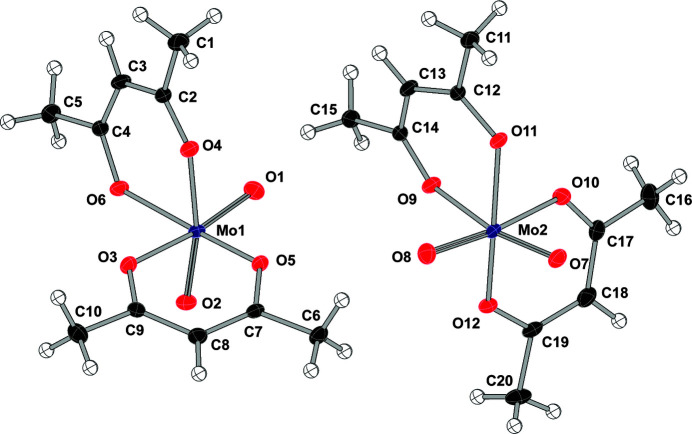
Displacement ellipsoid (50% probability) diagram of the two independent mol­ecules with the numbering scheme for the non-hydrogen atoms.

The asymmetric unit of the title compound contains two crystallographically independent *cis*-[MoO_2_(acac)_2_] mol­ec­ules, one each of the Δ and Λ forms (Fig. 1 ▸). The mol­ecular structure adopts a distorted octa­hedral arrangement around the Mo^VI^ atoms, with oxido ligands in a *cis* arrangement and oxido-molybdenum-oxido angles of 105.40 (4) and 105.59 (5)°. As observed previously (Krasochka, 1973 ▸; Kojić-Prodić *et al.*, 1974 ▸), the Mo—O bond distances *trans* to the molybdenum-oxygen double bonds are significantly lengthened [avg = 2.185 (5) Å] relative to the other molybdenum–oxygen distances [avg = 1.999 (11) Å] (see Table 1 ▸ for selected bond distances and angles). The four molybdenum oxygen distances for the doubly-bonded oxido ligands average 1.7012 (16) Å, in agreement with the average distance found for over 140 similar *cis*-dioxido molybdenum complexes in the Cambridge Structural Database (Groom *et al.*, 2016 ▸). These metrics are also in agreement with relatively narrow distribution of molybdenum–oxygen distances observed by Mayer (1988 ▸) for *cis*-dioxido complexes.

**Figure 2:**
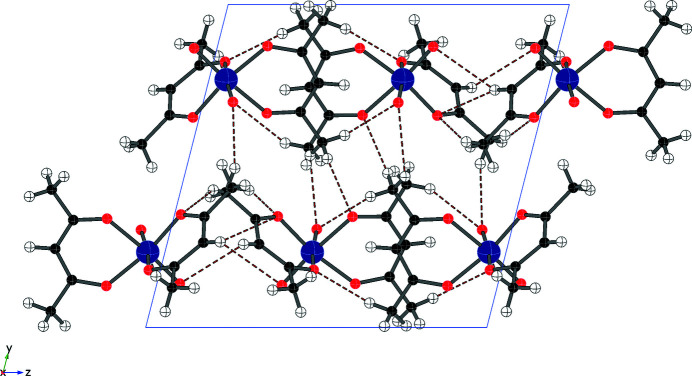
Packing diagram (viewed along *a*), showing extensive weak C—H⋯O contacts (red dotted lines) throughout the crystal structure.

All of the hydrogen-bonding contacts are weak C—H⋯O inter­actions with *D*⋯*A* distances between 3.3 and 3.5 Å (see Table 2 ▸ and Fig. 2 ▸). There are contacts between C—H atoms and all four of the oxido ligands, including two contacts to O1 and three contacts to O8. Additional C—H contacts are made to most of the acetyl­acetonate oxygen atoms as well.

## Synthesis and crystallization

The title compound was prepared using the *Inorganic Syntheses* procedure (Chakravorti & Bandyopadhyay, 1992 ▸) with some modifications adapted from Arnáiz (1995 ▸). A sample of 3.0 grams of ammonium *para*-molybdate was dissolved in 6.0 ml of 24%_wt_ aqueous ammonia. A syringe was used to add 7.0 ml of 2,4-penta­nedione with stirring. Concentrated nitric acid (5.0 ml) was added and the solution was stirred for 30 min. The product precipitated as a pale-yellow solid and was isolated by filtration and washed with deionized water (2 × 10 ml), followed by ethanol (1 × 10 ml), and diethyl ether (1 × 10 ml). Over multiple preparations the yield averaged around 90%. Characterization by ^1^H NMR and FTIR agrees with previously reported values (Chakravorti & Bandyopadhyay, 1992 ▸; Arnáiz, 1995 ▸).

Three different crystallization methods were utilized: slow evaporation from a concentrated solution in 2,4-penta­nedione, vapor diffusion (di­chloro­methane/diethyl ether), and layering (di­chloro­methane/diethyl ether) in a standard 5 mm NMR tube. All three methods produced crystals, but the highest quality crystals and those used in this study were produced from solvent layering.

## Refinement

Crystal data, data collection and structure refinement details are summarized in Table 3 ▸.

## Supplementary Material

Crystal structure: contains datablock(s) global, I. DOI: 10.1107/S2414314621007781/wm4150sup1.cif

Structure factors: contains datablock(s) I. DOI: 10.1107/S2414314621007781/wm4150Isup2.hkl

Supporting information file. DOI: 10.1107/S2414314621007781/wm4150Isup3.mol

CCDC reference: 2100177

Additional supporting information:  crystallographic information; 3D view; checkCIF report

## Figures and Tables

**Table 1:** Selected geometric parameters (Å, °)

Mo1—O1	1.7029 (9)	Mo2—O7	1.6996 (9)
Mo1—O2	1.7001 (9)	Mo2—O8	1.7021 (9)
Mo1—O3	2.1825 (8)	Mo2—O9	2.1808 (8)
Mo1—O4	2.1921 (8)	Mo2—O10	2.1848 (9)
Mo1—O5	2.0060 (8)	Mo2—O11	1.9898 (8)
Mo1—O6	1.9897 (8)	Mo2—O12	2.0106 (8)
			
O2—Mo1—O1	105.40 (4)	O7—Mo2—O8	105.59 (5)

**Table 2:** Hydrogen-bond geometry (Å, °)

*D*—H⋯*A*	*D*—H	H⋯*A*	*D*⋯*A*	*D*—H⋯*A*
C1—H1*A*⋯O8^i^	0.93 (1)	2.62 (2)	3.4834 (16)	155 (2)
C5—H5*B*⋯O10^ii^	0.98 (1)	2.50 (1)	3.4652 (15)	170 (2)
C6—H6*A*⋯O8	0.97 (1)	2.51 (2)	3.3894 (15)	151 (2)
C8—H8⋯O7^iii^	0.91 (1)	2.79 (2)	3.4071 (14)	126 (1)
C10—H10*A*⋯O6^iv^	0.95 (1)	2.68 (2)	3.3334 (15)	127 (1)
C10—H10*C*⋯O1^v^	0.97 (1)	2.50 (2)	3.3126 (15)	141 (2)
C11—H11*B*⋯O3^i^	0.99 (1)	2.48 (1)	3.4415 (14)	163 (2)
C15—H15*A*⋯O1^ii^	0.93 (1)	2.55 (2)	3.4592 (15)	167 (2)
C15—H15*C*⋯O4	0.96 (1)	2.53 (2)	3.4018 (15)	152 (2)
C16—H16*A*⋯O11^vi^	0.94 (1)	2.66 (2)	3.3127 (15)	128 (2)
C16—H16*B*⋯O8^vii^	0.96 (2)	2.52 (2)	3.3971 (17)	153 (2)
C18—H18⋯O2^viii^	0.92 (1)	2.82 (2)	3.4646 (15)	128 (1)

Symmetry codes: (i) 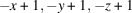 ; (ii) 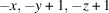 ; (iii) 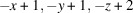 ; (iv) 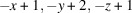 ; (v) 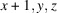 ; (vi) 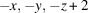 ; (vii) 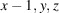 ; (viii) 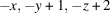 .

**Table 3:** Experimental details

Crystal data	
Chemical formula	[Mo(C_5_H_7_O_2_)_2_O_2_]
*M*_r_	326.15
Crystal system, space group	Triclinic, *P* 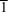
Temperature (K)	100
*a*, *b*, *c* (Å)	8.0111 (3), 12.4143 (4), 12.6847 (4)
α, β, γ (°)	75.649 (1), 89.272 (1), 87.072 (1)
*V* (Å^3^)	1220.56 (7)
*Z*	4
Radiation type	Mo *K*α
μ (mm^−1^)	1.09
Crystal size (mm)	0.28 × 0.22 × 0.14

Data collection	
Diffractometer	Bruker APEXII CCD
Absorption correction	Multi-scan (*SADABS*; Krause *et al.*, 2015 ▸)
*T*_min_, *T*_max_	0.676, 0.747
No. of measured, independent and observed [*I* > 2σ(*I*)] reflections	82074, 11855, 10556
*R*_int_	0.035
(sin θ/λ)_max_ (Å^−1^)	0.835

Refinement	
*R*[*F* ^2^ > 2σ(*F* ^2^)], *wR*(*F* ^2^), *S*	0.023, 0.061, 1.04
No. of reflections	11855
No. of parameters	391
No. of restraints	28
H-atom treatment	Only H-atom coordinates refined
Δρ_max_, Δρ_min_ (e Å^−3^)	1.19, −1.11

Computer programs: *APEX3* (Bruker, 2020 ▸), *SAINT* (Bruker, 2020 ▸), *SHELXT* (Sheldrick, 2015*a* ▸), *SHELXL* (Sheldrick, 2015*b* ▸), *CrystalMaker* (Palmer, 2019 ▸), *OLEX2* (Dolomanov *et al.*, 2009 ▸) and *publCIF* (Westrip, 2010 ▸).

## full crystallographic data

### Crystal data

**Table:**

[Mo(C_5_H_7_O_2_)_2_O_2_]	*Z* = 4
*M**_r_* = 326.15	*F*(000) = 656
Triclinic, *P*1	*D*_x_ = 1.775 Mg m^−^^3^
*a* = 8.0111 (3) Å	Mo *K*α radiation, λ = 0.71073 Å
*b* = 12.4143 (4) Å	Cell parameters from 9760 reflections
*c* = 12.6847 (4) Å	θ = 3.0–36.3°
α = 75.649 (1)°	µ = 1.09 mm^−^^1^
β = 89.272 (1)°	*T* = 100 K
γ = 87.072 (1)°	Block, yellow
*V* = 1220.56 (7) Å^3^	0.28 × 0.22 × 0.14 mm

### Data collection

**Table:**

Bruker APEXII CCD diffractometer	10556 reflections with *I* > 2σ(*I*)
φ and ω scans	*R*_int_ = 0.035
Absorption correction: multi-scan (SADABS; Krause *et al.*, 2015)	θ_max_ = 36.4°, θ_min_ = 1.7°
*T*_min_ = 0.676, *T*_max_ = 0.747	*h* = −13→13
82074 measured reflections	*k* = −20→20
11855 independent reflections	*l* = −21→21

### Refinement

**Table:**

Refinement on *F*^2^	28 restraints
Least-squares matrix: full	0 constraints
*R*[*F*^2^ > 2σ(*F*^2^)] = 0.023	Only H-atom coordinates refined
*wR*(*F*^2^) = 0.061	*w* = 1/[σ^2^(*F*_o_^2^) + (0.0307*P*)^2^ + 0.524*P*] where *P* = (*F*_o_^2^ + 2*F*_c_^2^)/3
*S* = 1.04	(Δ/σ)_max_ = 0.003
11855 reflections	Δρ_max_ = 1.19 e Å^−^^3^
391 parameters	Δρ_min_ = −1.11 e Å^−^^3^

### Special details

**Table:**

Refinement. All H atoms were located in a difference-Fourier map. Hydrogen atom positions were refined with C—H distances restrained to 0.98 (2) Å (CH_3_) or 0.95 (2) Å (ring C) and with *U*_iso_(H) = 1.5*U*_eq_(C) (methyl) or *U*_iso_(H) = 1.2*U*_eq_(C) (ring).

### Fractional atomic coordinates and isotropic or equivalent isotropic displacement parameters (Å^2^)

**Table:**

	*x*	*y*	*z*	*U*_iso_*/*U*_eq_	
Mo1	0.19729 (2)	0.76693 (2)	0.56793 (2)	0.01071 (2)	
O1	0.01992 (11)	0.69604 (7)	0.57195 (8)	0.01646 (15)	
O2	0.14825 (12)	0.86914 (7)	0.63247 (7)	0.01718 (15)	
O3	0.45296 (11)	0.82176 (7)	0.55088 (7)	0.01484 (14)	
O4	0.32120 (11)	0.65709 (7)	0.47653 (7)	0.01407 (14)	
O5	0.30012 (10)	0.65609 (7)	0.69609 (7)	0.01323 (13)	
O6	0.17058 (11)	0.86448 (7)	0.41831 (7)	0.01522 (14)	
C1	0.37036 (18)	0.55651 (10)	0.34261 (11)	0.0194 (2)	
H1A	0.408 (2)	0.5708 (16)	0.2707 (13)	0.029*	
H1B	0.457 (2)	0.5162 (15)	0.3884 (16)	0.029*	
H1C	0.286 (2)	0.5032 (14)	0.3486 (16)	0.029*	
C2	0.30151 (14)	0.65672 (9)	0.37795 (9)	0.01315 (17)	
C3	0.21648 (16)	0.74411 (10)	0.30091 (9)	0.01650 (19)	
H3	0.205 (2)	0.7355 (14)	0.2306 (12)	0.020*	
C4	0.16005 (14)	0.84288 (9)	0.32317 (9)	0.01329 (17)	
C5	0.08566 (17)	0.93714 (10)	0.23647 (10)	0.0183 (2)	
H5A	0.170 (2)	0.9885 (14)	0.2000 (15)	0.028*	
H5B	0.033 (2)	0.9095 (15)	0.1802 (14)	0.028*	
H5C	0.002 (2)	0.9801 (15)	0.2663 (16)	0.028*	
C6	0.42700 (16)	0.58188 (10)	0.86696 (9)	0.0170 (2)	
H6A	0.416 (2)	0.5069 (12)	0.8582 (16)	0.026*	
H6B	0.333 (2)	0.5953 (15)	0.9090 (15)	0.026*	
H6C	0.5274 (19)	0.5743 (15)	0.9049 (15)	0.026*	
C7	0.42379 (14)	0.66499 (9)	0.75910 (8)	0.01228 (16)	
C8	0.54477 (15)	0.74177 (10)	0.73039 (9)	0.01541 (18)	
H8	0.6317 (19)	0.7423 (14)	0.7754 (13)	0.018*	
C9	0.56069 (13)	0.81282 (9)	0.62473 (9)	0.01234 (17)	
C10	0.71393 (15)	0.87800 (10)	0.59652 (11)	0.0180 (2)	
H10A	0.689 (2)	0.9523 (12)	0.5569 (15)	0.027*	
H10B	0.776 (2)	0.8840 (15)	0.6561 (13)	0.027*	
H10C	0.788 (2)	0.8426 (15)	0.5532 (15)	0.027*	
Mo2	0.31234 (2)	0.23314 (2)	0.94971 (2)	0.01062 (2)	
O7	0.35933 (12)	0.13590 (7)	1.06697 (7)	0.01702 (15)	
O8	0.49058 (11)	0.30206 (7)	0.91316 (8)	0.01682 (15)	
O9	0.18784 (11)	0.33673 (7)	0.80520 (7)	0.01435 (14)	
O10	0.05669 (11)	0.17773 (7)	0.96628 (7)	0.01557 (15)	
O11	0.33915 (11)	0.12896 (7)	0.85341 (7)	0.01430 (14)	
O12	0.20855 (11)	0.34966 (7)	1.01892 (7)	0.01405 (14)	
C11	0.42605 (16)	0.05038 (9)	0.70948 (10)	0.01590 (19)	
H11A	0.506 (2)	0.0052 (14)	0.7626 (15)	0.024*	
H11B	0.481 (2)	0.0759 (15)	0.6381 (12)	0.024*	
H11C	0.338 (2)	0.0040 (14)	0.6990 (15)	0.024*	
C12	0.35213 (13)	0.14698 (9)	0.74799 (9)	0.01179 (16)	
C13	0.29618 (16)	0.24372 (9)	0.67571 (9)	0.01583 (19)	
H13	0.312 (2)	0.2494 (14)	0.6000 (11)	0.019*	
C14	0.20743 (13)	0.33250 (9)	0.70739 (9)	0.01210 (16)	
C15	0.13314 (16)	0.42696 (10)	0.62128 (10)	0.01670 (19)	
H15A	0.095 (2)	0.4053 (15)	0.5610 (13)	0.025*	
H15B	0.043 (2)	0.4672 (15)	0.6472 (16)	0.025*	
H15C	0.219 (2)	0.4773 (14)	0.5942 (15)	0.025*	
C16	−0.20236 (16)	0.12360 (11)	1.04748 (12)	0.0219 (2)	
H16A	−0.178 (2)	0.0492 (13)	1.0454 (17)	0.033*	
H16B	−0.280 (2)	0.1597 (16)	0.9911 (15)	0.033*	
H16C	−0.265 (2)	0.1222 (16)	1.1124 (14)	0.033*	
C17	−0.04876 (14)	0.18876 (9)	1.03822 (9)	0.01463 (18)	
C18	−0.03130 (16)	0.26312 (11)	1.10536 (10)	0.0182 (2)	
H18	−0.116 (2)	0.2651 (15)	1.1543 (14)	0.022*	
C19	0.08525 (14)	0.34274 (9)	1.08878 (9)	0.01445 (18)	
C20	0.07649 (18)	0.43275 (12)	1.14881 (11)	0.0220 (2)	
H20A	0.087 (3)	0.5039 (13)	1.1019 (16)	0.033*	
H20B	−0.027 (2)	0.4408 (16)	1.1843 (16)	0.033*	
H20C	0.163 (2)	0.4253 (17)	1.1994 (15)	0.033*	

### Atomic displacement parameters (Å^2^)

**Table:**

	*U*^11^	*U*^22^	*U*^33^	*U*^12^	*U*^13^	*U*^23^
Mo1	0.01212 (4)	0.01239 (4)	0.00833 (4)	−0.00019 (3)	−0.00151 (3)	−0.00391 (3)
O1	0.0145 (3)	0.0165 (4)	0.0190 (4)	−0.0011 (3)	−0.0029 (3)	−0.0055 (3)
O2	0.0220 (4)	0.0167 (4)	0.0150 (4)	−0.0011 (3)	0.0008 (3)	−0.0078 (3)
O3	0.0153 (3)	0.0185 (4)	0.0102 (3)	−0.0043 (3)	−0.0011 (3)	−0.0016 (3)
O4	0.0174 (4)	0.0143 (3)	0.0106 (3)	0.0010 (3)	−0.0004 (3)	−0.0037 (3)
O5	0.0147 (3)	0.0152 (3)	0.0094 (3)	−0.0023 (3)	−0.0017 (3)	−0.0018 (3)
O6	0.0229 (4)	0.0129 (3)	0.0099 (3)	0.0008 (3)	−0.0041 (3)	−0.0031 (3)
C1	0.0265 (6)	0.0172 (5)	0.0161 (5)	0.0006 (4)	0.0036 (4)	−0.0079 (4)
C2	0.0152 (4)	0.0140 (4)	0.0111 (4)	−0.0026 (3)	0.0016 (3)	−0.0043 (3)
C3	0.0244 (5)	0.0163 (5)	0.0096 (4)	−0.0001 (4)	−0.0026 (4)	−0.0048 (3)
C4	0.0157 (4)	0.0138 (4)	0.0102 (4)	−0.0028 (3)	−0.0027 (3)	−0.0020 (3)
C5	0.0240 (5)	0.0165 (5)	0.0127 (4)	−0.0004 (4)	−0.0066 (4)	0.0000 (4)
C6	0.0228 (5)	0.0171 (5)	0.0096 (4)	0.0007 (4)	−0.0027 (4)	−0.0004 (3)
C7	0.0156 (4)	0.0126 (4)	0.0090 (4)	0.0017 (3)	−0.0015 (3)	−0.0037 (3)
C8	0.0176 (5)	0.0159 (4)	0.0126 (4)	−0.0016 (4)	−0.0050 (4)	−0.0029 (3)
C9	0.0133 (4)	0.0114 (4)	0.0134 (4)	0.0000 (3)	−0.0004 (3)	−0.0051 (3)
C10	0.0146 (5)	0.0172 (5)	0.0234 (6)	−0.0040 (4)	0.0003 (4)	−0.0070 (4)
Mo2	0.01235 (4)	0.01240 (4)	0.00711 (4)	0.00003 (3)	0.00073 (3)	−0.00262 (3)
O7	0.0226 (4)	0.0168 (4)	0.0105 (3)	−0.0003 (3)	−0.0010 (3)	−0.0013 (3)
O8	0.0153 (4)	0.0165 (4)	0.0181 (4)	−0.0016 (3)	0.0027 (3)	−0.0033 (3)
O9	0.0188 (4)	0.0146 (3)	0.0095 (3)	0.0024 (3)	−0.0003 (3)	−0.0034 (3)
O10	0.0151 (3)	0.0185 (4)	0.0147 (4)	−0.0040 (3)	0.0028 (3)	−0.0066 (3)
O11	0.0214 (4)	0.0131 (3)	0.0083 (3)	0.0013 (3)	0.0016 (3)	−0.0029 (2)
O12	0.0161 (3)	0.0159 (3)	0.0115 (3)	−0.0007 (3)	0.0016 (3)	−0.0062 (3)
C11	0.0213 (5)	0.0137 (4)	0.0135 (4)	0.0010 (4)	0.0031 (4)	−0.0052 (3)
C12	0.0132 (4)	0.0124 (4)	0.0102 (4)	−0.0018 (3)	0.0025 (3)	−0.0037 (3)
C13	0.0244 (5)	0.0145 (4)	0.0085 (4)	0.0012 (4)	0.0011 (4)	−0.0031 (3)
C14	0.0146 (4)	0.0116 (4)	0.0100 (4)	−0.0021 (3)	−0.0006 (3)	−0.0020 (3)
C15	0.0233 (5)	0.0129 (4)	0.0127 (4)	−0.0002 (4)	−0.0046 (4)	−0.0009 (3)
C16	0.0159 (5)	0.0220 (5)	0.0248 (6)	−0.0044 (4)	0.0025 (4)	0.0002 (4)
C17	0.0139 (4)	0.0151 (4)	0.0127 (4)	0.0001 (3)	0.0004 (3)	0.0007 (3)
C18	0.0185 (5)	0.0222 (5)	0.0141 (5)	0.0002 (4)	0.0065 (4)	−0.0054 (4)
C19	0.0173 (4)	0.0173 (4)	0.0091 (4)	0.0039 (4)	−0.0006 (3)	−0.0050 (3)
C20	0.0263 (6)	0.0251 (6)	0.0181 (5)	0.0048 (5)	−0.0002 (4)	−0.0135 (5)

### Geometric parameters (Å, º)

**Table:**

Mo1—O1	1.7029 (9)	Mo2—O7	1.6996 (9)
Mo1—O2	1.7001 (9)	Mo2—O8	1.7021 (9)
Mo1—O3	2.1825 (8)	Mo2—O9	2.1808 (8)
Mo1—O4	2.1921 (8)	Mo2—O10	2.1848 (9)
Mo1—O5	2.0060 (8)	Mo2—O11	1.9898 (8)
Mo1—O6	1.9897 (8)	Mo2—O12	2.0106 (8)
O3—C9	1.2624 (13)	O9—C14	1.2623 (13)
O4—C2	1.2635 (13)	O10—C17	1.2632 (14)
O5—C7	1.3067 (13)	O11—C12	1.3036 (13)
O6—C4	1.3041 (13)	O12—C19	1.3104 (14)
C1—H1A	0.933 (14)	C11—H11A	0.984 (14)
C1—H1B	0.947 (15)	C11—H11B	0.988 (14)
C1—H1C	0.957 (14)	C11—H11C	0.962 (14)
C1—C2	1.5010 (16)	C11—C12	1.4961 (15)
C2—C3	1.4183 (16)	C12—C13	1.3761 (16)
C3—H3	0.931 (14)	C13—H13	0.953 (14)
C3—C4	1.3782 (16)	C13—C14	1.4191 (16)
C4—C5	1.4971 (16)	C14—C15	1.4942 (16)
C5—H5A	0.983 (14)	C15—H15A	0.932 (14)
C5—H5B	0.975 (14)	C15—H15B	0.961 (14)
C5—H5C	0.968 (15)	C15—H15C	0.957 (14)
C6—H6A	0.973 (14)	C16—H16A	0.941 (14)
C6—H6B	0.946 (14)	C16—H16B	0.961 (15)
C6—H6C	0.931 (14)	C16—H16C	0.955 (15)
C6—C7	1.4952 (16)	C16—C17	1.4944 (17)
C7—C8	1.3762 (16)	C17—C18	1.4152 (17)
C8—H8	0.907 (14)	C18—H18	0.917 (14)
C8—C9	1.4184 (16)	C18—C19	1.3709 (17)
C9—C10	1.4955 (16)	C19—C20	1.4986 (17)
C10—H10A	0.948 (14)	C20—H20A	0.942 (15)
C10—H10B	0.931 (14)	C20—H20B	0.950 (15)
C10—H10C	0.965 (14)	C20—H20C	0.935 (15)
			
O1—Mo1—O3	165.66 (4)	O7—Mo2—O8	105.59 (5)
O1—Mo1—O4	89.35 (4)	O7—Mo2—O9	164.58 (4)
O1—Mo1—O5	93.60 (4)	O7—Mo2—O10	87.98 (4)
O1—Mo1—O6	98.03 (4)	O7—Mo2—O11	95.53 (4)
O2—Mo1—O1	105.40 (4)	O7—Mo2—O12	96.98 (4)
O2—Mo1—O3	88.56 (4)	O8—Mo2—O9	89.81 (4)
O2—Mo1—O4	165.18 (4)	O8—Mo2—O10	166.15 (4)
O2—Mo1—O5	97.16 (4)	O8—Mo2—O11	97.67 (4)
O2—Mo1—O6	95.35 (4)	O8—Mo2—O12	94.14 (4)
O3—Mo1—O4	76.80 (3)	O9—Mo2—O10	76.68 (3)
O5—Mo1—O3	81.17 (3)	O11—Mo2—O9	81.38 (3)
O5—Mo1—O4	82.99 (3)	O11—Mo2—O10	83.47 (3)
O6—Mo1—O3	83.63 (3)	O11—Mo2—O12	159.82 (4)
O6—Mo1—O4	80.95 (3)	O12—Mo2—O9	82.36 (3)
O6—Mo1—O5	160.01 (4)	O12—Mo2—O10	81.22 (3)
C9—O3—Mo1	127.94 (7)	C14—O9—Mo2	128.23 (7)
C2—O4—Mo1	128.41 (7)	C17—O10—Mo2	127.20 (8)
C7—O5—Mo1	130.40 (7)	C12—O11—Mo2	131.47 (7)
C4—O6—Mo1	132.43 (7)	C19—O12—Mo2	129.74 (7)
H1A—C1—H1B	108.5 (17)	H11A—C11—H11B	109.9 (16)
H1A—C1—H1C	105.8 (17)	H11A—C11—H11C	108.6 (15)
H1B—C1—H1C	103.1 (16)	H11B—C11—H11C	106.5 (15)
C2—C1—H1A	115.0 (12)	C12—C11—H11A	111.4 (11)
C2—C1—H1B	113.3 (13)	C12—C11—H11B	110.9 (10)
C2—C1—H1C	110.2 (12)	C12—C11—H11C	109.4 (11)
O4—C2—C1	117.22 (10)	O11—C12—C11	114.43 (9)
O4—C2—C3	123.67 (10)	O11—C12—C13	124.15 (10)
C3—C2—C1	119.10 (10)	C13—C12—C11	121.35 (10)
C2—C3—H3	117.7 (11)	C12—C13—H13	118.0 (10)
C4—C3—C2	123.42 (10)	C12—C13—C14	123.58 (10)
C4—C3—H3	118.8 (11)	C14—C13—H13	118.3 (10)
O6—C4—C3	124.17 (10)	O9—C14—C13	123.53 (10)
O6—C4—C5	114.20 (10)	O9—C14—C15	117.53 (10)
C3—C4—C5	121.60 (10)	C13—C14—C15	118.93 (10)
C4—C5—H5A	112.3 (11)	C14—C15—H15A	113.4 (12)
C4—C5—H5B	110.8 (11)	C14—C15—H15B	113.3 (12)
C4—C5—H5C	111.2 (12)	C14—C15—H15C	108.1 (11)
H5A—C5—H5B	106.9 (16)	H15A—C15—H15B	107.6 (16)
H5A—C5—H5C	107.7 (16)	H15A—C15—H15C	105.6 (16)
H5B—C5—H5C	107.8 (16)	H15B—C15—H15C	108.5 (15)
H6A—C6—H6B	105.2 (16)	H16A—C16—H16B	110.9 (18)
H6A—C6—H6C	101.7 (16)	H16A—C16—H16C	107.2 (17)
H6B—C6—H6C	112.9 (17)	H16B—C16—H16C	102.8 (17)
C7—C6—H6A	110.9 (12)	C17—C16—H16A	112.3 (12)
C7—C6—H6B	110.0 (12)	C17—C16—H16B	109.7 (12)
C7—C6—H6C	115.2 (12)	C17—C16—H16C	113.5 (12)
O5—C7—C6	114.28 (10)	O10—C17—C16	117.36 (11)
O5—C7—C8	124.26 (10)	O10—C17—C18	123.12 (11)
C8—C7—C6	121.45 (10)	C18—C17—C16	119.44 (11)
C7—C8—H8	121.1 (11)	C17—C18—H18	115.8 (12)
C7—C8—C9	123.91 (10)	C19—C18—C17	124.01 (10)
C9—C8—H8	114.2 (11)	C19—C18—H18	119.2 (11)
O3—C9—C8	123.05 (10)	O12—C19—C18	124.32 (10)
O3—C9—C10	117.50 (10)	O12—C19—C20	114.39 (11)
C8—C9—C10	119.42 (10)	C18—C19—C20	121.27 (11)
C9—C10—H10A	112.2 (12)	C19—C20—H20A	112.1 (13)
C9—C10—H10B	114.7 (12)	C19—C20—H20B	114.9 (12)
C9—C10—H10C	109.6 (11)	C19—C20—H20C	112.8 (13)
H10A—C10—H10B	105.2 (16)	H20A—C20—H20B	102.8 (17)
H10A—C10—H10C	108.5 (16)	H20A—C20—H20C	104.7 (18)
H10B—C10—H10C	106.2 (16)	H20B—C20—H20C	108.6 (18)
			
Mo1—O3—C9—C8	14.91 (16)	Mo2—O9—C14—C13	11.74 (16)
Mo1—O3—C9—C10	−167.23 (8)	Mo2—O9—C14—C15	−167.95 (8)
Mo1—O4—C2—C1	−165.78 (8)	Mo2—O10—C17—C16	−167.33 (8)
Mo1—O4—C2—C3	13.14 (16)	Mo2—O10—C17—C18	15.86 (16)
Mo1—O5—C7—C6	159.72 (8)	Mo2—O11—C12—C11	160.69 (8)
Mo1—O5—C7—C8	−21.55 (16)	Mo2—O11—C12—C13	−22.43 (17)
Mo1—O6—C4—C3	−21.18 (18)	Mo2—O12—C19—C18	−20.31 (17)
Mo1—O6—C4—C5	160.91 (8)	Mo2—O12—C19—C20	161.61 (8)
O4—C2—C3—C4	6.18 (19)	O10—C17—C18—C19	10.74 (19)
O5—C7—C8—C9	−7.08 (18)	O11—C12—C13—C14	−4.41 (19)
C1—C2—C3—C4	−174.93 (11)	C11—C12—C13—C14	172.26 (11)
C2—C3—C4—O6	−3.75 (19)	C12—C13—C14—O9	8.31 (19)
C2—C3—C4—C5	174.01 (11)	C12—C13—C14—C15	−172.00 (11)
C6—C7—C8—C9	171.56 (11)	C16—C17—C18—C19	−166.01 (12)
C7—C8—C9—O3	9.00 (18)	C17—C18—C19—O12	−10.0 (2)
C7—C8—C9—C10	−168.82 (11)	C17—C18—C19—C20	167.99 (12)

### Hydrogen-bond geometry (Å, º)

**Table:**

*D*—H···*A*	*D*—H	H···*A*	*D*···*A*	*D*—H···*A*
C1—H1*A*···O8^i^	0.93 (1)	2.62 (2)	3.4834 (16)	155 (2)
C5—H5*B*···O10^ii^	0.98 (1)	2.50 (1)	3.4652 (15)	170 (2)
C6—H6*A*···O8	0.97 (1)	2.51 (2)	3.3894 (15)	151 (2)
C8—H8···O7^iii^	0.91 (1)	2.79 (2)	3.4071 (14)	126 (1)
C10—H10*A*···O6^iv^	0.95 (1)	2.68 (2)	3.3334 (15)	127 (1)
C10—H10*C*···O1^v^	0.97 (1)	2.50 (2)	3.3126 (15)	141 (2)
C11—H11*B*···O3^i^	0.99 (1)	2.48 (1)	3.4415 (14)	163 (2)
C15—H15*A*···O1^ii^	0.93 (1)	2.55 (2)	3.4592 (15)	167 (2)
C15—H15*C*···O4	0.96 (1)	2.53 (2)	3.4018 (15)	152 (2)
C16—H16*A*···O11^vi^	0.94 (1)	2.66 (2)	3.3127 (15)	128 (2)
C16—H16*B*···O8^vii^	0.96 (2)	2.52 (2)	3.3971 (17)	153 (2)
C18—H18···O2^viii^	0.92 (1)	2.82 (2)	3.4646 (15)	128 (1)

Symmetry codes: (i) −*x*+1, −*y*+1, −*z*+1; (ii) −*x*, −*y*+1, −*z*+1; (iii) −*x*+1, −*y*+1, −*z*+2; (iv) −*x*+1, −*y*+2, −*z*+1; (v) *x*+1, *y*, *z*; (vi) −*x*, −*y*, −*z*+2; (vii) *x*−1, *y*, *z*; (viii) −*x*, −*y*+1, −*z*+2.
